# Half-time evaluation of a new 4-year Ph.D. program in nursing and midwifery at the University of Gondar, Ethiopia

**DOI:** 10.1080/16549716.2021.1905304

**Published:** 2021-08-17

**Authors:** Kerstin Erlandsson, Helena Lindgren, Lena Wettergren, Alemayehu Mekuriaw, Marta Berta, Mengstu Melkamu, Tewodros Seyoum, Solomon Hailemeskel, Abebaw Jember, Mohammed Hassen, Mignote Gebrie, Biftu Geda, Kassahun Gelaye, Sewunet Habtamo Mekonnen, Salomon Mekonnen Abebe, Kyllike Christensson

**Affiliations:** a Women’s and Children’s Health, Karolinska Institutet, Sweden; b Institute of Public Health, College of Medicine and Health Science, University of Gondar, Ethiopia; c School of Midwifery, College of Medicine and Health Science, University of Gondar, Ethiopia; d School of Nursing, College of Medicine and Health Science, University of Gondar, Ethiopia; e Department of Nursing, College of Health and Medical Science, Meda Welabu University, Ethiopia; f Health Sciences, Addis Ababa University by College of Health Science, Addis Ababa University, Ethiopia

**Keywords:** Stig Wall, Nursing, midwifery, capacity building, half-time evaluation, Ph.D. education

## Abstract

A new four-year Ph.D. programme in nursing and midwifery, the first of its kind in Ethiopia, was started in 2018/2019 at the University of Gondar when eight doctoral students in nursing and midwifery entered the program. We who have been involved see this as an appropriate time to evaluate what has been accomplished to date and to look toward future possibilities. Our aim in carrying out such an evaluation and presenting our findings is in part to determine if similar programs might be developed in other similar settings and in part to learn what modifications to the present program might be considered. The key elements of a questionnaire survey with closed and open response alternatives were based on the content, structure and learning outcomes of the home university Ph.D. programme as described in the curriculum. The questionnaire responses captured changes that would be needed to maintain a fully satisfactory programme that blends onsite instruction and online access to faculty resulting in a twenty-first century blended Ph.D. programme. Improved dialogue between the home university faculty and the external supervisors is needed. The programme can provide a career pathway that midwifery and nursing educators can follow in their own country rather than having to leave to study in another country. The findings provide insight into the feasibility of extending similar Ph.D. programmes to other parts of East Africa and with the SDG 5 in mind with an increased focus on women leadership. The justification for this initiative is to meet the need for more nursing and midwifery faculty who can provide quality midwifery and nursing education in East African countries. Retention of these professionals will help to deal with the shortage of healthcare personnel and will provide better care for the general population.

## Background

In an era where the shortage of midwives and nurses is of universal concern, career path and higher level programmes for diploma-holding midwives and nurses are needed. In East African countries meeting the demand for a skilled workforce requires national strategies for postgraduate recruitment and retention of graduates within these countries. A key element of such strategies is to provide for home-based Ph.D. programs [1]. The value of the Ph.D. for career advancement of nurses and midwives had already been described in 2001 [2]. Now in the twenty-first century, the need to provide higher level Nursing and Midwifery education has been recognized, a level of education not provided when independent nursing and midwifery schools were standard. During the past 40 years, those who were engaged in teaching in nursing and midwifery programs also began to become researchers [2,3]. It became evident that some of these teachers would begin to face the same demands faced by university faculty in other fields, that is publication in scientific journals, attendance at research conferences, seeking research funding, and taking on responsibility for academic leadership. Nursing and Midwifery faculty who combine teaching with research will lead to improved quality of education in these fields. This in turn can be expected over a longer time to lead to better patient care [3,4]. In the context of the global shortage of nurse and midwife graduates with Ph.Ds, scaling up nursing and midwifery education has been recommended by the WHO to increase the capacity of national healthcare systems [5,6]. Following the introduction of net-based Ph.D. programmes in the 1990s, researchers in Europe, USA, Middle East, Australia, South Africa [1,4,7–9] began to evaluate such so-called distance Ph.D. programs and found that the graduates were well positioned to provide leadership in the areas of science, practice, and education. We looked for, but did not find, any evaluations of capacity building initiatives providing for Ph.D. programs in East Africa. We did, however, recognize the same need as the researchers cited above [1,3,6], a need to be able to recruit and retain Ph.D. candidates in nursing and midwifery in East Africa.

We, faculty and researchers at the University of Gondar, hereafter called the ‘home university’ in Ethiopia and the Karolinska Institutet, the ‘external university’ in Sweden describe here the process by which a Ph.D. program was created at the University of Gondar. We developed a curriculum and pedagogic approaches for supervision of the research component of the planned Ph.D. Development, approval, and implementation of the program was a joint process carried out by the two partner universities. The Memorandum of Understanding (MoU) for this programme was signed with the enrolment of Ph.D. students in 2018/2019. The students were selected based on professional and academic merits. The project plans for the students were presented to and approved by a research committee consisting of researchers from the two universities. The strategy set up by the home university research committee was that the home university had sufficient capacity for running the Ph.D. programme independently without technical support from the external university by 2024. The first Ph.D. candidates, hereafter called ‘candidates’ are expected to graduate in 2022 after defending their completed dissertations. The dissertations will be similar in structure to dissertations at the external university in Sweden and will consist of a framing section and the three to four scientific publications either already published or to be accepted for publication in peer-review journals.

The courses in the planned Ph.D. programme included sections dealing with medical science – epidemiology, biostatistics, qualitative and quantitative research, review of the scientific literature and identification of current medical issues. In addition, attention would be given to philosophical and ethical aspects of advanced medical care. During the first two years, the external lecturers/supervisors managed the courses online and when possible face to face with the candidates at the home university. Before the COVID-19 pandemic, it was possible for the external supervisors to visit the home university for six weeks per year with financial support from each of the two partner universities. Between the visits, two home- and two external supervisors assigned to each candidate advised the respective candidates online. The researchers and supervisors were faculty and researchers with an average of 20 years of experience in leadership and supervision in research and education. The external university researchers were all female, and the home university researchers were all male. After the outbreak of the COVID-19 pandemic, all courses and supervision were provided online only.

The Ph.D. programme in nursing and midwifery introduced at College of Medicine and Health Science, University of Gondar, Ethiopia is the first of its kind in East Africa. The programme was created by the combined efforts of College of Medicine and Health Science, University of Gondar, Ethiopia and Women’s and Children’s Health, Karolinska Institutet, Solna, Sweden with four assigned researchers from each of the two partner universities. The researchers are here sharing their experiences after analyzing the eight Ph.D. candidates’ half-time evaluations of a blended Ph.D. programme. The external lecturers/supervisors, hereafter called external supervisors, lectured and supervised Ph.D. candidates enrolled and financed by the home university.

This program, begun in 2018/2019, reached its half-way point in 2020/2021. We decided to carry out a half-time evaluation of this capacity building initiative in June 2020 when concerns were raised in connection with the spread of the Covid 19 virus. We who had been involved saw this as an appropriate time to evaluate what has been accomplished to date and to look toward future improvements. We set out to examine how successfully the Ph.D. candidates were able to adhere to this blended online and face-to-face Ph.D. programme. Our hope was that this review might indicate what kind of improvements might be made in the second half of the Ph.D. program and also help in determining the feasibility for creating similar programs elsewhere.

## Methodology

### Design

**Figure 1.**
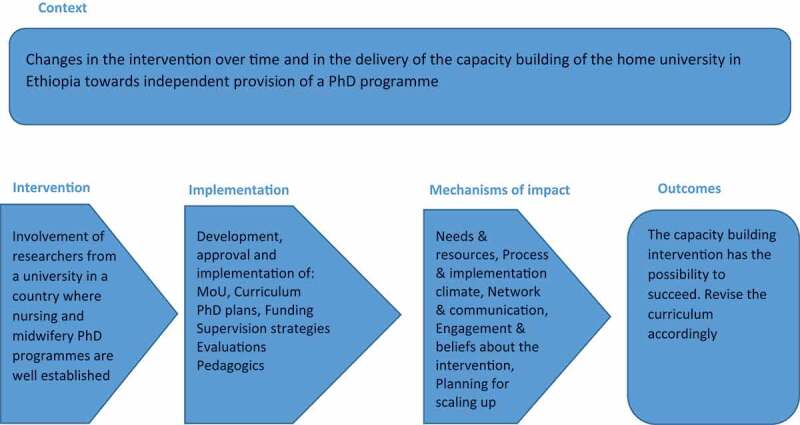
Framework for describing the Ph.D. program in this paper inspired by Moore and coworkers [10]

The evaluation of the capacity building represented by the new Ph.D. programme was performed in a manner based on Moore et al. [10]. (Please see Figure 1).

Intervention, Implementation, and  Context  of this project was in line with the foreign Swedish policy on gender equality [11] and the Ethiopia human resources for health strategic plan [12] and have already been described in the Background section. The open and closed response alternatives in the data collection instruments will be presented in the Method section. The results are presented in the Mechanism of Impact and Outcome section below [10,13]. Research ethics principles were followed and considered [14].

### Inclusion of participants

The eight candidates enrolled in the Ph.D. programme were included in this half-time evaluation. The Ph.D. candidates were four midwives and four nurses with an average of 10 years of working experience and 5–10 years as nursing and midwifery faculty at universities in nearby Ethiopian regions. The nursing and midwifery faculty hold Masters degree in nursing and midwifery before being appointed to faculty in the Gondar University nursing and midwifery Ph.D. programme. The Ph.D. programme is leading to a Ph.D. degree in nursing or midwifery from an Ethiopian university. Two of the candidates were women.

### Data collection instrument

**Figure 2.**
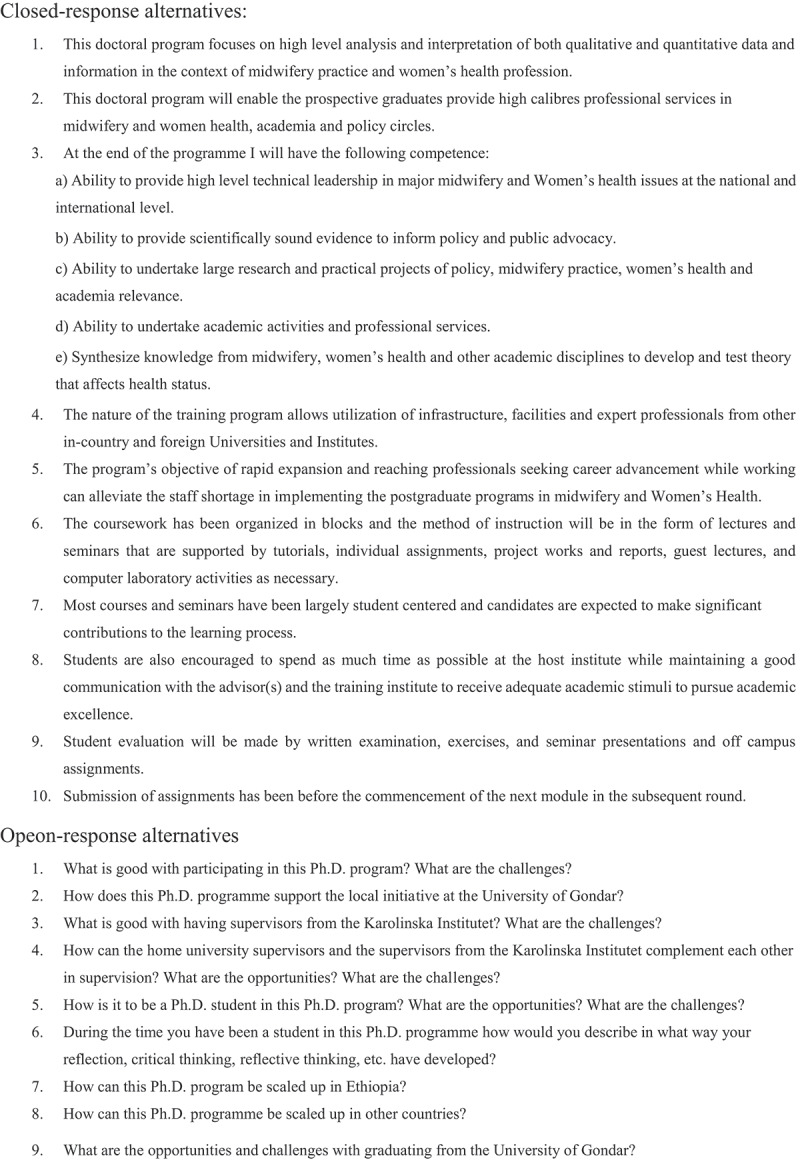
Questionnaire for closed and open response alternatives

A questionnaire was developed [13]. The closed-response alternatives were presented in English, based on statements, not questions, on content, structure and learning outcomes of the home university Ph.D. programme as described in the curriculum (Figure 2).

The level of agreement with the statements was rated in a Likert scale [13]. The 5-point Likert scale was comprising the alternatives 1) strongly disagree, 2) disagree, 3) neither agree nor disagree, 4) agree, and 5) strongly agree. After each statement, there was space for comments. The open response alternatives were provided in a separate section at the end of the questionnaire encouraging the students to write freely as much as required to answer the open questions (Figure 2). The closed and open response alternatives were pilot tested by home university and external university lecturers/supervisors.

### Data collection procedure

Data were collected in June 2020 during the lockdown due to the Covid-19 pandemic.

The eight candidates participating in the doctorate at the time of answering the questionnaires in their home environment signed an informed consent, and the questionnaires were then sent by e-mail to a third person who compiled and removed any identifying material from the questionnaires before sending them to HL and KE for analysis [13].

## Analysis

The researchers HL and KE actively set their pre-understanding aside when analysing the data. The Likert scale alternatives 1) strongly disagree and 2) disagree were collated and classified as ‘lack of agreement with the statements’ while 4) agree and 5) strongly agree were collated and classified as ‘agreed with the statements’. The number 3 was classified as ‘neither agree nor disagree’. The answers to the Likert scale alternatives in number (n) and percentage (%) are presented first in the Mechanism of impact section.

In the last part of the Mechanism of impact section the results of the analysis of open-ended questions are presented. The analysis of the open-ended questions followed guidelines for carrying out content analysis [15]. The responses to the questionnaire consisting of 30 pages of written text were first read and re-read and then divided according to similarities and differences in the texts and finally inserted under five emerging themes [15].

## Results

### Mechanisms of impact

#### Results from the closed response alternatives

Overall, the candidates agreed with the content and structure of the Ph.D. programme. The eight candidates (100%) agreed to all but two statements in the questionnaire. Two candidates (25%) out of eight neither agreed nor disagreed with the following two statements.

- The nature of the training program allows utilization of infrastructure, facilities and expert professionals from other in-country and foreign Universities and Institutes.
- Students are also encouraged to spend as much time as possible at the host institute while maintaining a good communication with the advisor(s) and the training institute to receive adequate academic stimuli to pursue academic excellence.

They commented that they wanted to meet the home university supervisor more often. They also wanted to be supported by professionals from other disciplines. They commented that they would like to meet all the supervisors together, that is those from the external university as well as the home.

#### Results from analysis of the open-ended questions

*Theme 1. Supervision*

**Table:** 

***Theme*** *Supervision*	***Subtheme***
	*External supervisors were perceived as a guarantee for fulfilment of the Ph.D. programme*
	*The candidates had to balance the different views on research designs between public health and the nursing/midwifery supervisors*
	*The different perspectives of the supervisors could be enriching to the candidate*

*Subtheme 1: External supervisors were perceived as a guarantee for fulfilment of the Ph.D. programme*

The implementation of the joint Ph.D. education programme was perceived as insurance for the accomplishment of the Ph.D. programme within the timeframe. The outbreak of the Covid-19 pandemic however brought up concerns regarding continuation of the support from external supervisors (6): *They came for advisory trips at least three times a year, they responded immediately so we could in this way achieve the goals. The* Covid-19 *pandemic blocked the advisory visits and hampered all that we had planned regarding data gathering (6).*

*Subtheme 2: The candidates had to balance the different views on research designs between public health and midwifery/nursing supervisors*

The difference between the field of public health and the fields of nursing and midwifery had to be balanced by the candidates. The home university public health supervisors provided hands-on support to the candidates and advocated for large-scale population-based data gatherings whilst the external supervisors were more focused on making small-scale qualitative and quantitative studies that directly impacted clinical nursing and midwifery care. … .*the philosophy of nursing/midwifery and public health are quite different and it is reflected in the way you plan your research project (3).*

*Subtheme 3. The different perspectives of the supervisors could be enriching to the candidate*

As long as each group of supervisors communicated their group perspective on public health versus nursing and midwifery research to the other group these different views were seen as enriching by the candidates. The supervisors complemented each other: *One of the beauties of having supervisors from the home university and the external university is that they have different research experiences and culture. So, having them involved with each other is a nice complement and will benefit the Ph.D. candidates (4)*. The candidates could facilitate meetings between the home university and the external supervisors and these joint supervision sessions were resource and could make the supervision team communicate needs, and in such a way they could complement each other in benefit to the candidate.

*Theme 2: A stressful time period in life*

**Table:** 

***Theme*** *A stressful time period in live*	***Subtheme***
	*A journey with ups and downs along the way.*
	*Having Ph.D. students as lecturers favours University of Gondar and the candidate*

*Subtheme 1. A journey with ups and downs along the way*

In the first year of the Ph.D. programme, the first four candidates’ engagement was high with completion of all courses. Personally and professionally the candidates expressed that they developed a full commitment in their striving to complete the Ph.D. programme. Their engagement in education and research changed their professional perspectives in terms of reflective and critical thinking and also influenced their personal beliefs about the adherence to the Ph.D. programme. *My understanding, critical thinking, interpreting, and the way of handling health-related questions have changed. I am always proud of the program when I see myself in a position that understands things easily, critically, and from different perspectives (6)*. The candidates agreed that a high-level education programme produced strategic, reflective, and critical thinkers, who developed their scientific ability and that was an opportunity worth the journey: *I think it has a lot of opportunities for me, getting in-depth knowledge about research plus the quality of midwifery and nursing and third I will become a fellow Ph.D. holder to others with the same level of Ph.D. (2)*. A particular journey was the process of data gathering for the projects. The candidates lived at the home university; they left their families behind in their home city and this affected their daily life and perhaps state of mind. The financial constraints for accommodating and gathering data were seen as a concern by the Ph.D. candidates. The budget provided by the home university was not large enough to cover the costs. According to the candidates, in addition to the financial challenges, there were difficulties following the timeline for the research projects, due to the pandemic situation and security issues: *We were planning to collect our data in June for tool validation but as you know, we now sit in our home for more than three months (due to Covid-19 lock down) and the second factor influencing the implementation is the lack of financial resources and security (8)*. One suggestion was to make a plan for how to meet the challenges from pandemics and potential security instabilities.

*Subtheme 2. Having Ph.D. students as lecturers favours University of Gondar and the candidate*

The first-year candidates gave lectures to nursing and midwifery students on nursing and midwifery in theory and practice. The lectures were provided part time by the students while they also took their own classes and conducted research. The opportunities of graduating from the home university, not from a foreign university were perceived by the candidates as a potential aid making it easier for the Ph.D. programme to be scaled up in Ethiopia and other East African countries. After graduating the candidates were planning to leave for their hometown universities to live and work there. The data gathering took place near their home universities. In such a way the quality of education and practice was enhanced at the University of Gondar: *The nursing and midwifery Ph.D. education program will enhance the evidence-based practice at the Gondar comprehensive specialized hospital (8).*

Being part of the Ph.D. program was seen as providing a good opportunity for the candidate to be able to develop a career as a tutor and leader in nursing and midwifery in higher education. *After graduation, I will work at my home university. I will teach and advise my undergraduate and postgraduate students and I will change the work with other institutions in the area of student advice and teaching (4)*. The pedagogic strategies with student-centered learning in the Ph.D. programme had in turn influenced the candidates’ engagement in teaching practices.

*Theme 3: Online lecturing and communication*

**Table:** 

***Theme*** *Online lecturing and communication*	***Subtheme***
	*Covid-19 forced us to progress in online communication*
	*This modality with online and onsite supervision and programmes has come to stay also after the pandemic*

*Sub-theme 1. Covid-19 forced us to progress in online communication*

The Covid-19 pandemic required replacing communication via face-to-face lecturing and supervision with Zoom and e-mail correspondence. Using these communication approaches was difficult especially in the beginning in part because of the weak internet connectivity: *For successful completion of this programme and successful expansion in Ethiopia and other countries, it might be helpful with good connectivity. It is not easy with weak internet connectivity (5)*. The online sessions, when the connectivity was good, provided some opportunities for helping the candidates learn how to communicate and network through Zoom in a foreign language using break out rooms for group discussions, networking and presenting.

*Subtheme 2. This modality with online and onsite supervision and programmes has come to stay also after the pandemic*

The candidates emphasized the importance of access to online learning resources, tools for analysis of qualitative and quantitative data, books, and scientific articles. They realized there will be more networking in conferences, education, programmes and collaborations online in the future after the Covid-19 pandemic is over. *Taking the standpoint forced on us by the Covid-19 pandemic experience will make us use the internet in research and education much more, sharing the Ph.D. studies with other scholars at the national and global university level (7)*. One suggestion was to include in the curriculum how you access online literature, databases, tools for analysis and networking online at conferences.

*Theme 5: Planning for the scaling up*

The demand for high-level nursing and midwifery education could be met in Ethiopia if plans can be formulated for a scaling up of Ph.D. programmes at other centres if support can also be found. The graduates from the home university could support other universities initiating career pathways for nurses and midwives by initiating Ph.D. opportunities for them. The candidates emphasised the need for required collaboration with the Ministry of Education and Ministry of Health and supervisors at a home university willing to collaborate with supervisors from abroad. *In Ethiopia, there are seven universities at an equal stage and these universities could become collaborating partners with an external university. By following the same principle, you apply at Gondar University in Ethiopia you can expand it to other countries. One thing that programme needs is commitment and passion from the home universities (4)*. Collaboration between foreign and home universities, with a relevant budget for the investment from the home university was seen by the candidates as the best idea to prevent brain-drain from home countries with a tremendous need to retain Ph.D. holders in the country to produce a quality midwifery and nursing workforce: *Investing in a Ph.D. education programme in nursing and midwifery may decrease the brain drain instead of funding Ph.D. scholarships abroad (1)*. The candidates stressed the need for curriculum review: *I think the curriculum should be revised and incorporate sharing of experiences through attending international meetings online* (7).

## Outcome and priorities

This capacity building initiative for midwifery and nursing educators has possibilities for succeeding. The suggested priorities based on the half-time evaluation are to revise the curriculum including sections on how to, a) meet the challenges from pandemics and potential security instabilities, b) access online literature, databases, tools for analysis and networking online at conferences, c) work in a timely manner on the Ph.D. proposal, assignments, manuscripts, and the dissertation proposal, d) seek funds enabled by the provision of sufficient internet connectivity, e) supervise the Ph.D. candidates with supervisors from the home university and the external university, f) capacity build each other’s competences as supervisors in joint supervision sessions with the candidate throughout the Ph.D. education programme, g) in future programmes, encourage the selection of women to become Ph.D. candidates and women to become teachers and researchers in the Ph.D. programme at the home university.

## Discussion

The descriptions of this capacity building initiative for nursing and midwifery educators in terms of a Ph.D. programme are presented as part of a process evaluation [10] and have captured changes in the initiative that would be needed to further progress of this pioneer Ph.D. programme in nursing and midwifery in Ethiopia toward reaching higher scholarly outcomes. The candidates at the home university expressed a desire to expand their research knowledge as part of the challenge of fulfilling their *personal and professional goals* in line with published qualitative descriptions of Ph.D. candidates who have written about the same challenges, telling readers that it was worth the journey as the candidates gained personal and professional fulfillment while working towards the Ph.D. degree [16]. The Sustainable development goal 5 [17] points to the need for women in leadership positions [18]. In the Ph.D. programme at the home university, only two candidates were female. In the findings, nothing was shared about time- and stress-management from the female home university candidates in contrast with findings in a USA-based interview study among Ph.D. candidates where the female candidates expressed how they balanced all of their responsibilities associated with work, family, and school. The *financial constraints* that were raised in this process evaluation by the candidates at the home university in Ethiopia were concerns related to research productivity and programme progression, related to funding constraints during doctoral study, concerns similar to findings in other studies [19] but not related to workload due to being a lecturer or in relation to constrained home situations while separated from their families. To meet the shortage of nursing and midwifery educators, providing quality nursing and midwifery education in Ethiopia and other East African countries, doctoral programs must be provided wherever possible. Eventually, these programs will benefit the entire population of Ethiopia or any other country [20]. In Ethiopia, the home university has a lot to gain from investing in providing a Ph.D. program in nursing and midwifery educators. The Ph.D. holders will devote their lives to helping thousands of students and/or patients when they complete their education [5]. With this in mind, one useful marketing strategy described in the literature was raising funds from various sources. Starck (2015) argue that adequate student and programmatic resources could help the Ph.D. candidates get some full time, uninterrupted study for completion of the doctoral thesis [5]. The Ph.D. holders will become faculty, remain in the country, and will then educate students and care for patients during their careers. In line with previous findings on *international collaborations*, the candidates at home universities would benefit from discussing issues that occur as part of the Ph.D. process with their supervisors as suggested by other authors [9]. Taking part in national and international conferences and exchange programmes online would encourage the candidates to continue their journey and could be encouraged by exchange of experts from foreign universities [21]. Exchange of experts can strengthen education and research links between countries [22] as pointed out by the candidates in this process evaluation. Physical visits had according to the candidates at the home university a value that cannot fully be replaced by the virtual meetings. The candidates at the home university recognised that the different *supervision and pedagogical approaches* at the home university and by the Swedish supervisors had a positive influence on students [23]. Different approaches in pedagogy and research interests make us jointly realise the importance of well-designed and well-prepared Ph.D. programmes [24]. The curriculum will now be revised for the two remaining years to come and actions will be taken for a post pandemic exchange programme between the University of Gondar and the Karolinska Institutet to further strengthen education and research links.

## Strength and limitations of this process evaluation

This Ph.D. programme has been half time evaluated and presented in a manner inspired by Moore and co-author’s treatment of process evaluation [10]. The modified method frame enabled us to describe this capacity builidng intitiative. Ethical approval was obtained by the University of Gondar Ethical board for the Ph.D. projects in this capacity building initiative [13,14,25]. The paper lacks coherence to some degree because of our handling of reporting guidelines for improvement studies from the Equator Network, and to the COREQ checklist [25]. We have focused on communicating to the audience how the evaluation was conducted, and the analysis section refers to the answers to the questionnaire used by the eight Ph.D. candidates at the half-time evaluation with Likert scale and qualitative data without claiming any reliability or depth in analysis as required for an original mixed method survey. With these limitations in mind, we have followed ethics for all involved in this half-time evaluation inspired by the research process of Moore and co-workers [10,13]. Describing a capacity building initiative using process evaluation as a frame for the presentation we conclude was helpful.

## Conclusion and clinical implications

The descriptions in the result section captured changes that would be needed in this capacity building initiative for nursing and midwifery educators, to progress towards scholarly outcomes. The programme can provide a career pathway for nursing and midwifery educators who want to stay and work in their own country to provide both education and care in the future. The findings provide insight into the feasibility of expanding similar capacity building initiatives for nursing and midwifery educators in terms of Ph.D. programmes to other parts of Ethiopia and other East African countries. The justification for this is to meet the shortage of educators providing quality midwifery and nursing education in Ethiopia and other East African countries and in turn, meet the shortage of health workforce providing quality care to the population of a country. To promote sustainable development goal with respect to goal 5 that highlights the need for women in leadership positions we recommend the examination of the situation for female Ph.D. candidates at home universities and what types of motivation will help to involve female candidates in Ph.D. programmes.

## Paper context

In the context of the global shortage of nursing and midwifery educators, scaling up nursing and midwifery Ph.D. programmes has been recommended to address the sustainability and capacity of national healthcare systems. This paper adds to the topic candidates’ adherence to a blended online and face-to-face Ph.D. programme at Gondar University, Ethiopia, supported by an external university. This study provides valuable insight into the feasibility of expanding similar Ph.D. programmes to similar settings.
